# Extended Environmental Multimedia Modeling System (EEMMS) with Analytic Hierarchy Process for Dual Evaluation of Energy Consumption and Pollutants in Solid Waste

**DOI:** 10.3390/toxics13100878

**Published:** 2025-10-15

**Authors:** Jing Yuan, Heng Wang, Meifeng Chen

**Affiliations:** 1 Department of Civil Engineering, Tongling University, Tongling 244000, China; 2 Department of Civil Engineering, Manitoba University, Winnipeg, MB R3T2N2, Canada; 3 Binjiang Institute of Artificial Intelligence, Zhejiang University of Technology, Hangzhou 310014, China; 4 Engineering Research Institute, Anhui University of Technology, Ma’anshan 243002, China

**Keywords:** landfill leachate, Extended Environmental Multimedia Modeling System (EEMMS), MODFLOW, Monte Carlo Method (MCM), energy consumption

## Abstract

The dual assessment of environmental risks and energy consumption of solid waste is crucial for ensuring environmental safety and energy consumption management. Using risk assessment tools to inform best management practices for reclamation is very important. In this paper, a former Extended Environmental Multimedia Modeling System (EEMMS) combined with the Monte Carlo Method (MCM) of risk assessment was further used for exploring the fate and migration of pollutant leakage in the CFSWMA landfill. Specifically, MODFLOW combined with the EEMMS–MCM system has been applied using Biochemical Oxygen Demand (BOD) as a typical indicator to model the behavior of leachate components. An EEMMS–MCM integrated risk assessment for a 20-year period was conducted. The case study of BOD emissions from the CFSWMA landfill shows that even the leachate did not have a serious impact on Canadian territory during the 20 years; however, non-sorption chemicals are mainly affected by the groundwater flow, whereas sorption chemicals are affected by the partition coefficient (or sorption). Further, this study introduces energy consumption factors such as soil and surface water bodies, and constructs an integrated dual assessment framework for the environmental risks and energy consumption of pollutants. In summary, by integrating the EEMMS pollutant migration model with an environmental risk and energy consumption assessment, a dual assessment of environmental risks and energy consumption is achieved.

## 1. Introduction

In the past 40 years, developed countries in Europe and the United States have carried out a series of studies on the basic theories and methods of landfill, soil, and groundwater pollution, including source, process, mechanism, effect, risk, and prediction [1]. Landfills are an important way to reduce water pollution [2]. Leachate from landfills infiltrates underground, causing serious water pollution and endangering people’s lives and health [3]. In the processes of a landfill, some leachate will appear, and the concentration of leachate is very high. If it is not controlled in time, leachate will enter the surface, which will bring a large amount of pollution to the water quality and even lead to soil and groundwater pollution [4]. The decision to attempt landfill remediation is one of the most difficult management issues for municipal and state agencies, as contaminated soil and groundwater have been the subject of much concern in the last decade, with many concurrent assessments and cleanups [5].

The dual assessment of environmental risks and energy consumption of solid waste is crucial for ensuring environmental safety and energy consumption management. Traditional waste management methods often lack a comprehensive evaluation of multimedia and multilevel impacts, making it difficult to accurately predict the long-term effects of waste on the environment and health. For instance, traditional single-media models, unable to capture cross-media interactions, can only explain less than 30% of the variation in pollutant exposure risks. Therefore, researchers have proposed a multilevel and multimedia modeling framework to more comprehensively simulate and assess the migration, transformation, and accumulation processes of solid waste in different environmental media. The development of multimedia, multilevel modeling has made great progress recently, especially with the development of the microscopic molecular mechanism, multiple media transmission mechanism, multiple interface allocation mechanism, multiscale model, and so on [6,7,8,9,10,11,12]. At the same time, studies have been able to analyze multiscale pollutant source/channel-sink and form/dose-acceptor relationships, and have established a pollution source database, biological toxicity database, and environmental reference system [13]. Furthermore, pollutant migration risk, ecological risk, and health risk assessment methods have been developed based on environmental background and investigation studies of the landfill, soil, and groundwater [14,15]. Detailed theories, methods and technical principles of environmental pollution prevention and control have been formed, and the effective control methods of soil–plant and soil–groundwater pollutants based on interface behavior regulation have been developed [16,17]. All these laid a theoretical and methodological foundation for the establishment of environmental quality standards for contaminated soil and the design of new remediation technologies [18,19].

With the emergence and rapid development of computational modeling, Giraud applied computer technologies to hydrogeological numerical modeling for the first time in the 1960s [20]. With the use of computational modeling, the results obtained are more accurate and, furthermore, the application process is more convenient [21,22]. A wide range of computational modeling techniques to simulate groundwater flow and mass transport issues have been developed during the past few decades [23]. The most widely used of these programs is the MODFLOW model, which simulates three-dimensional groundwater flow using the finite-difference method [24]. The technologists conducted a lot of in-depth research and made qualitative or quantitative analyses of the results, which provided a solid theoretical basis for the preassessment of the environmental impact of landfills on groundwater and contributed many research ideas and a theoretical basis for the subsequent research work [25,26,27].

Therefore, it is necessary to combine computational modeling with environmental pollution assessment and risk assessment to find a better environmental assessment and remediation tool. Specifically, researchers focus on the traditional pollutants, the single pollution process and its ecological effects, and the emerging pollutants and their combined pollution process and health effects [28]. For example, Yuan et al. began with a single physical, chemical, or biological process and mechanism and developed a multimedia, multi-interface, and multi-process coupling mechanism [2]. In terms of analysis and monitoring, the process developed from the content analysis of a single pollutant into the monitoring and analysis of multiple coexisting pollutants, from a microscopic point source analysis to a macroscopic multisource, multiscale three-dimensional intelligent soil monitoring [29,30]. In terms of remediation technology and utilization, the single remediation technology expanded to a multi-technology coupling system, from source control, process blocking, purification and restoration, and safe utilization to the ecological development of an integrated governance and redevelopment mode [31,32,33]. In terms of risk and management, intelligent risk management integrates remote sensing, the Internet of Things, and big data, incorporating factors from health risks to ecological risks, from pollution source barriers to process control [34,35,36].

However, there are obvious limitations in risk assessment and risk management. A key challenge is to identify major anthropogenic sources of pollution and assess their downstream environmental and socioeconomic impacts [37,38]. Such assessments are needed, for example, to identify cost-effective remedies that can mitigate the adverse effects of sources of pollution [38]. However, the impact of inland sources on downstream recipients depends on different conditions in different water systems, including groundwater, rivers, lakes, and reservoirs, along the pollutant transport routes connecting the source and recipient [39]. A reliable assessment may also need to consider that conditions may differ in each system due to the heterogeneity of space–time hydrology, hydrogeology, and geochemistry, and that transport may occur in multiple stages (e.g., dissolved or suspended materials) [32,40]. In addition, most pollutants are affected by conversion and deceleration processes acting along flow paths, which can contribute to the natural attenuation of pollution loads. These processes include dispersion, adsorption, dissolution–precipitation, and chemical reactions [41,42]. Due to the complexity and interactions of different processes in different water systems, the major challenge requires an understanding of the net impact of inland sources of pollution on recipients. For example, Rabbani et al. [43] studied the application of phase change multiphase flow on homogeneous porous media in soil remediation.

Among the multimedia models, the EEMMS model (Extended Environmental Multimedia Modeling System (EEMMS)) proposed by Jing Yuan et al. is a typical representative example. This model achieves the simulation of the multidimensional and multistage coordinated migration of pollutants in space and time by constructing the interaction processes among various environmental media such as air, water, soil, and sediment [36,44,45]. The Analytic Hierarchy Process (AHP) has revolutionized the decision-making model for waste management by quantifying the trade-off relationship between energy efficiency and environmental safety. Lee and Tan (2023) developed an AHP framework for a municipal solid waste system, which reduced the total energy consumption of the system by 18% through optimizing facility locations [46]. The Hierarchical Multimedia Modeling Framework (HMMF) integrates energy flow analysis and pollutant migration models to achieve the dual assessment of environmental risks and energy consumption [47,48,49].

Parameters like Biochemical Oxygen Demand (BOD), Chemical Oxygen Demand (COD), and nutrient levels (e.g., ammonia, phosphorus) are used in data analysis to diagnose the type and severity of water pollution. BOD indicates biodegradable organic matter, while COD provides a broader measure of oxidizable pollutants, and their ratio helps assess treatment feasibility. In modeling, these data are crucial for calibrating and validating mathematical simulations of environmental systems [50]. They directly influence predictions by quantifying the rates of chemical and biological processes, such as oxygen depletion in a river or nutrient removal in a treatment plant. Ultimately, the accuracy of this input data determines the model’s reliability in forecasting outcomes, assessing risks, and evaluating the effectiveness of different management strategies, such as optimizing a treatment process or predicting the environmental impact of a pollutant discharge.

In conclusion, current research mainly focuses on the evaluation of risks from a single pollutant or a single-level AHP technical solution. There is a lack of a systematic research framework that integrates pollutant migration simulation, AHP hierarchical management, environmental risk analysis, and energy consumption evaluation [51]. This study will construct an integrated framework for the dual assessment of environmental risks and energy consumption. By integrating the EEMMS pollutant migration model and the hierarchical analysis method and combining it with environmental risk assessment, this study can achieve the dual assessment of environmental risks and energy consumption. This research is expected to enhance the scientific understanding and systematisms of environmental pollution control and provide a theoretical basis and decision-making support for the formulation of efficient and green environmental governance strategies [52].

## 2. Methodology

The methodology includes the following: (1) simulating the spatial–temporal migration process of pollutants in the multimedia model based on EEMMS; (2) constructing a hierarchical risk index and assessment system centered on the behavioral characteristics and environmental sensitivity of pollutants; and (3) introducing energy consumption factors such as soil and groundwater bodies to achieve the dual evaluation of environmental risks and pollution control benefits.

### 2.1. EEMMS Model

The environmental multimedia modeling approach has been widely used for simulating contaminant transport in unsaturated and groundwater media. Yet a commonly used environmental multimedia model that has only first-order accuracy may introduce considerable numerical errors under certain circumstances (Table 1). This study presents a finite element Extended Environmental Multimedia Modeling Analysis System (EEMMS) for the unsaturated landfill and groundwater case studies with an incorporation of finite element numerical analysis.

In this study, Visual MODFLOW is the most complete and user-friendly modeling environment for practical applications in three-dimensional groundwater flow and contaminant transport simulation [53]. Visual MODFLOW 6 (2017) was used to predict the CFSWMA landfill’s groundwater flow direction and the 20-year contaminant flow and concentration migration. Then, an Extended Environmental Multimedia Modeling System (EEMMS) was further used to evaluate the groundwater transport risk from the CFSWMA landfill. The EEMMS was set up to demonstrate the transport risk of leachates in groundwater. The governing equation of the EEMMS model is as follows [36,44,45]; a representative components BOD (Biochemical Oxygen Demand) has been chosen for the modeling. The movement of the flow and transport of BOD in an aquifer and the spatial and temporal distribution of the BOD concentrations have been presented in this study.

As part of EEMMS, for comprehensive risk assessment, the Monte Carlo Method will be used to consider uncertain parameters in the EEMMS model, and the values of these parameters will be generated by the cumulative distribution function and the corresponding probability density function [54]. The detailed method has been described in the authors’ previous articles [36,44,45]. After certain sets of random samples for each parameter are generated, the distribution of predicted concentrations for each grid square will be calculated by the EEMMS model [55]. 

**Figure 1:**
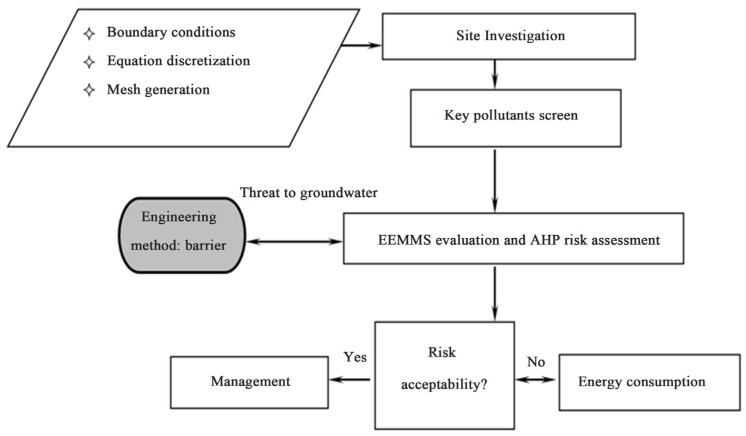
Assessment and EEMMS prediction and AHP assessment process of typical pollutants in contaminated sites.

The detailed assessment and prediction management and the AHP assessment process of typical pollutants in contaminated sites are summarized in Figure 1. For the chosen site, site investigation should be carried out first, including determining boundary conditions, what kind of equation discretization should be used, what kind of grid generation method should be used, and so on. Secondly, major representative pollutants will be selected according to the groundwater pollution investigation results, and then EEMMS will be used to improve the evaluation and its risk assessment. Finally, the risk of these major pollutants to the surrounding environment will be checked to see if they are acceptable, and if not, necessary remediation or treatment will be carried out. If the assessment shows a potential hazard to groundwater, engineering measures such as barriers (Figure 1) should be considered.

### 2.2. Case Study—CFSWMA Landfill

To meet the challenge, the Franklin County Solid Waste Authority (CFSWMA) was chosen as a typical landfill site [56]. Specifically, the current impact of the landfill was assessed using the EEMMS model to predict the spatial extent of its pollutant plume and the future environmental impact using AHP.

#### 2.2.1. Site Information

**Figure 2:**
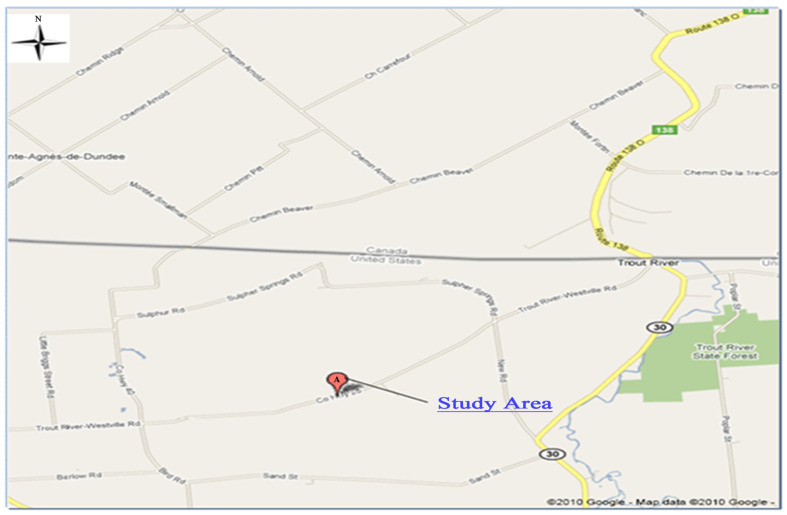
Site location and information.

Figure 2 provides an overview of the study site named the CFSWMA landfill (44.56 N, 74.17 W), which is located in Canada’s east coast region. The CFSWMA landfill was built in the late 1980s and early 1990s. The landfill covers a total area of 32.8 square miles (85.0 square kilometers). The northern border of the town is the international border between the United States and Quebec, Canada. The landfill is surrounded by Beaver Creek and the Trout River, and finally reaches the Chateauguay River. The Trout River is a very important river on the Canadian border at the junction of New York Route 30 and County Route 20. The landfill was chosen primarily because of the possible transboundary impacts that an expanded landfill might have, particularly on groundwater systems around Quebec, and the need to assess contaminant transport in the groundwater. If the expansion of the CFSWMA landfill violates a long-standing pollutant agreement in transboundary waters between Canada and the United States, millions of dollars will be spent on cleanup efforts and alternative water supplies for affected households in the future.

#### 2.2.2. Site Hydrological Characteristics and Contaminant Sources

The site’s hydrological parameters include hydrological properties and pore media properties. Hydrologic properties include water dynamic viscosity, water density, average recharge rate, water saturation, etc. Different media properties include hydraulic conductivity, porosity, bulk density, surface ground elevation, groundwater elevation, and groundwater gradient and direction. Hydrological characteristics of the site are shown in Table 2, which provides detailed input information for the model.

Table 3 shows the water table in four directions at the site. The data are mainly from the field investigation report [56].

The first layer has an elevation of 250 ft and the second layer has an elevation of 246 ft and a slope of 1% to the south [56]. The three layers contain two different soil materials. The first layer is upper glacial till. The material is brown sand, silt, and medium coarse gravel. The second layer is glacial till. The material is brown silt and fine to medium gravel. The third layer is overlying deposits under the spreading area, mainly composed of material of glacial and proglacial origin, with basal lodgment till. The first layer appears to be at a higher elevation to the north of the site and a lower elevation to the south, while the third layer appears to be at a higher elevation to the south, meaning that at this level the groundwater flows into Canadian territory. It also suggests that Quebec’s side of the border is at risk of potential leachate at the landfill when groundwater flows are considered. Groundwater movement mainly occurs from high head to low head. It was found that the groundwater flow mainly flows towards the northern Quebec border [57].

Groundwater quality pollution factors were analyzed using the Analytic Hierarchy Process (AHP). Various pollutants were classified into three major categories: organic pollution, inorganic pollution, and heavy metal pollution. Weights were set to construct a judgment matrix based on the environmental hazards of pollutants. Heavy metal pollution is significantly more important than organic pollution, heavy metal pollution is strongly more important than inorganic pollution, and organic pollution is slightly more important than inorganic pollution. Thus, Table 4 is obtained. Column normalization and consistency tests were conducted on the judgment matrix, from which Table 5 is obtained.

A judgment matrix was constructed and subsequently normalized (Table 4), followed by a consistency verification (Table 5) to ensure the robustness and reliability of the weighting process. The analytical results demonstrate that heavy metals exhibit the highest environmental risk, succeeded by organic pollutants (e.g., BOD), whereas inorganic conservative anions present the lowest relative risk level. However, BOD was selected as the main simulation indicator because it exhibits moderate sorption (K_d_) and a measurable decay rate, effectively representing both migration and natural attenuation processes in landfill leachate. Unlike sorptive heavy metals with high retardation and limited mobility, or conservative anions with low K_d_ and negligible decay that move almost identically with groundwater, BOD reflects an intermediate and environmentally responsive behavior.

Through AHP-based weighting analysis, heavy metals were identified as having the highest environmental risk, followed by organic pollutants (BOD) and inorganic anions. Although BOD’s intrinsic toxicity is lower than that of heavy metals, its mobility and biodegradability make it an effective indicator for assessing pollutant transport and risk distribution. Comparative simulations further show that BOD generates a moderate plume size, balancing migration and degradation, thereby serving as a representative parameter for predicting pollutant fate and evaluating landfill environmental risks.

So, in this case study, further model validation of concentrations more specifically related to Biochemical Oxygen Demand (BOD) will be typically considered in the concentration transport simulation section. The characteristics of site pollution sources are shown in Table 6.

#### 2.2.3. Model Result

Therefore, based on old data from the CFSWMA Landfill Environmental Impact Statement [36,56], the CFSWMA landfill was designed for a capacity of approximately 100,000 tons and is expected to reach a maximum capacity of 125,000 tons per year in 2024. It is estimated that the CFSWMA landfill processes 11,655 gallons of garbage per day per year [36,46]. If the other five batteries work at the same time in the future, the rate could reach 69,930 gallons per day. The Franklin County Sanitary Landfill will continue to produce gas for more than 50 years of garbage decomposition. Landfill emissions are mainly composed of BOD, methane, carbon dioxide, and nitrogen. To examine the risk of future BOD to Canadian territory, an initial BOD concentration of 1000 mg/L was assumed [58]. BOD represents the amount of oxygen required by microorganisms to degrade organic matter in water. BOD is soluble in water and follows a water transport pattern.

**Figure 3:**
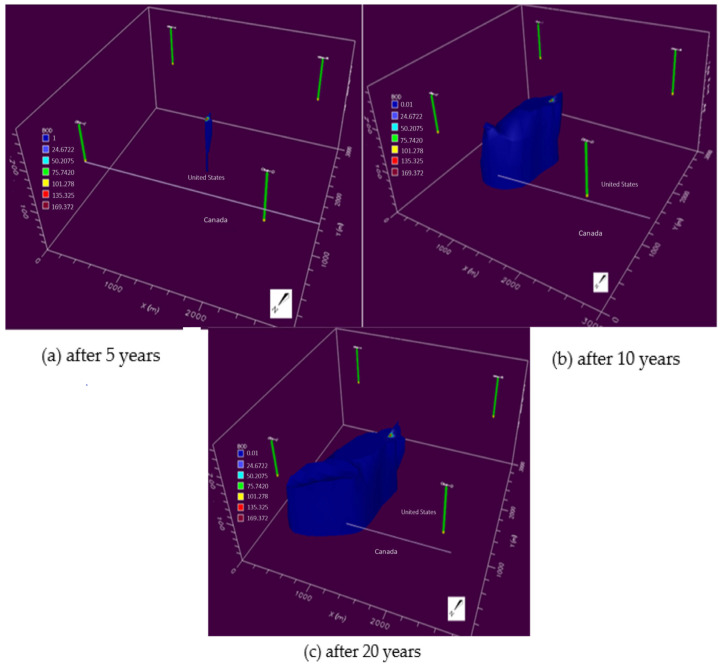
Distribution of the concentration of BOD (mg/L) escaped from CFSWMA landfill leachate (**a**) after 5 years, (**b**) after 10 years, and (**c**) after 20 years.

Suppose leachate from a landfill is accidentally discharged into groundwater. In this case, BOD can be used as a tracer for the fate of groundwater leachate components at 5, 10, and 20 years after the accident. Based on modeling parameters and assumptions, the BOD distribution simulation of the third layer is shown in Figure 3 when the transportation at 5, 10, and 20 years is considered, respectively.

Figure 3 shows the distribution of BOD after 5 years at a minimum concentration of 0.1 mg/L. The results show that the chemicals in the leachate (including BOD) migrate vertically near the bottom of the layer. Figure 3 shows that after 10 years, the BOD will reach the Canadian border. Since transportation of potential pollutants is primarily affected by groundwater flow rates, pollutants will be transported downstream when they reach the Canadian border. As can be seen from Figure 3, over a period of 20 years, the same distribution pattern can be found, but by the time they arrive in Canada, concentrations are between 0.01 mg/L and 0.1 mg/L.

## 3. Results

The leachate from the landfill has a high concentration of dissolved components. Due to different leachate production rates, ages, and waste compositions, concentrations of these chemicals are usually ten to several hundred times higher than those measured at reference monitoring sites. An effective comprehensive risk assessment can provide a systematic procedure for predicting potential risks to the environment.

**Figure 4:**
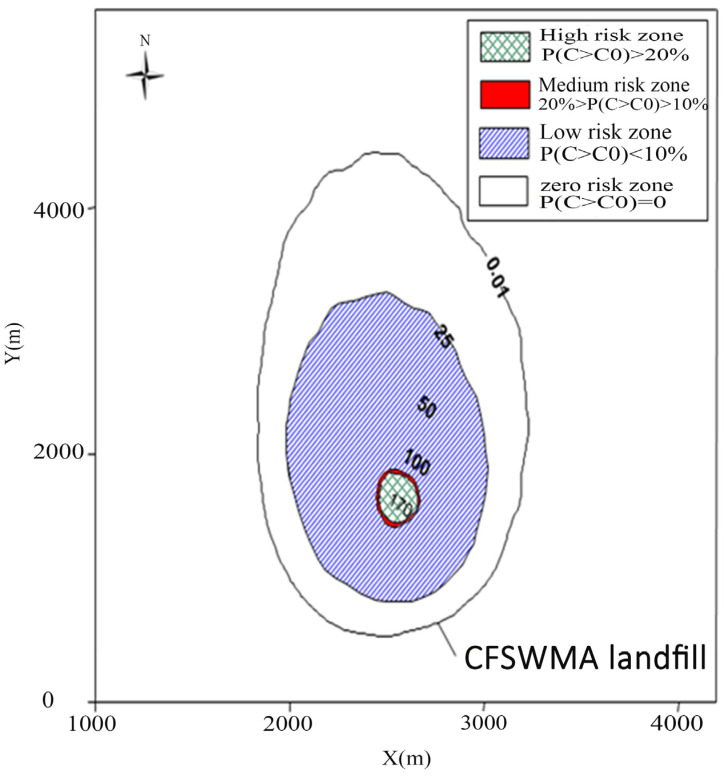
BOD mean concentration distribution and risk scale map for exceeding the criterion 25 mg/L in 20 years.

Figure 4 show the small area of the high- and medium-risk zone and part of the low-risk zone around the CFSWMA landfill in the 20-year period after the spill. Figure 4 shows the scale of severity associated with the probabilities of exceeding the standard (25 mg/L) [59]. As shown in Figure 4, the high-risk zone, which has a larger severity than the BOD standard (25 mg/L), is located within an area of the percentile concentration level (*P*(*C* > *C_standard_*) > 20%) around the CFSWMA landfill site. The inside shaded area indicates a percentile concentration level lower than the BOD standard (25 mg/L), which indicates a medium-risk level (0% < *P*(*C* > *C_standard_*) < 20%) and a low-risk level (P(C > *C_standard_*) < 10%). Figure 4 show that the high- and medium-risk zone is between 10 m to 100 m away from the source, which is a very thin area with a mean concentration of around 25 mg/L. The low-risk zone covers an area up to 1000 m away from the source in an area outside the medium-risk zone, which has a concentration between 25 to 0.01 mg/L. The rest of the area will have a zero-probability compared with the BOD concentration criteria. It could be understood that there is no BOD impact at the Canadian border after 20 years of transportation. This risk assessment method is simple and effective and can intuitively rank the possible risk areas associated with the potential pollutants in groundwater, providing a more comprehensive assessment of landfill leachate leakage risk for decision-makers.

**Figure 5:**
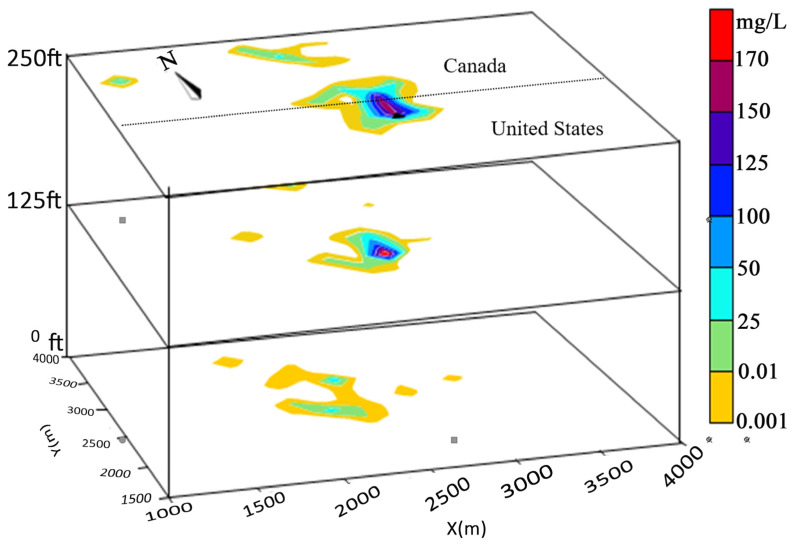
The 3D visualization of the BOD dispersion modeling results for 20 years.

Figure 5 shows BOD transport at 0, 125, and 250 ft after 20 years. The distribution of BOD is generally northward due to water flow, but concentrations of BOD accumulate higher near the CFSWMA landfill due to continued discharge. Figure 5 shows the modeling results for three layers over a 20-year period, which indicates that the plume reaches the northern boundary of Canadian territory horizontally and reaches a depth of 125 ft and 0 ft vertically, respectively.

The BOD concentration of the CFSWMA landfill was between 0.01 and 0.1 mg/L within 2000 m near the landfill source in the first layer. In the other two layers, concentrations are increasingly low, less than 0.1 mg/L, and at 0 ft, the distribution is more random, mainly due to lower near-bottom flow rates and complex bed friction effects.

Based on the above risk impact assessment, by introducing energy consumption fac-tors such as soil and groundwater bodies, the management process can be quantified for specific cases, helping to optimize the disposal plan, as shown in Table 7 below.

According to the multi-related party weight allocation model for energy consumption [63], the formula is as follows:

W*_total_* = w*_1_* × (HQ + CR × 10^6^) + w*_2_* × (E*_total_*/benchmark energy consumption) (1)

Here, the health allocation includes cancer risk plus non-cancer risk. The cancer risk (CR) in this case is 1.5 × 10^−7^, and the non-cancer risk (HQ) is calculated as follows: HQ = ADD/minimum pollution circle BOD pollution concentration value (mg/kg·day) = 0.00014/0.01 = 0.014. If CR and HQ are less than 1, it indicates no significant risk. The energy consumption allocation calculation formula is as follows: energy consumption allocation = E*_total_*/benchmark energy consumption, where E*_total_* takes the unit energy consumption × area from Table 7, and the benchmark energy consumption is 300 kWh/ton. The final calculated energy consumption and risk comprehensive index is as follows:

W*_total_* = 0.6 × (0.014 + 0.15) + 0.4 × (241/300) = 0.6 × 0.164 + 0.4 × 0.803 = 0.099 + 0.321 = 0.42 < 1, which indicates no significant risk. This case shows that when W*_total_* is less than 1, it indicates that the energy consumption is still acceptable.

## 4. Discussion

**Figure 6:**
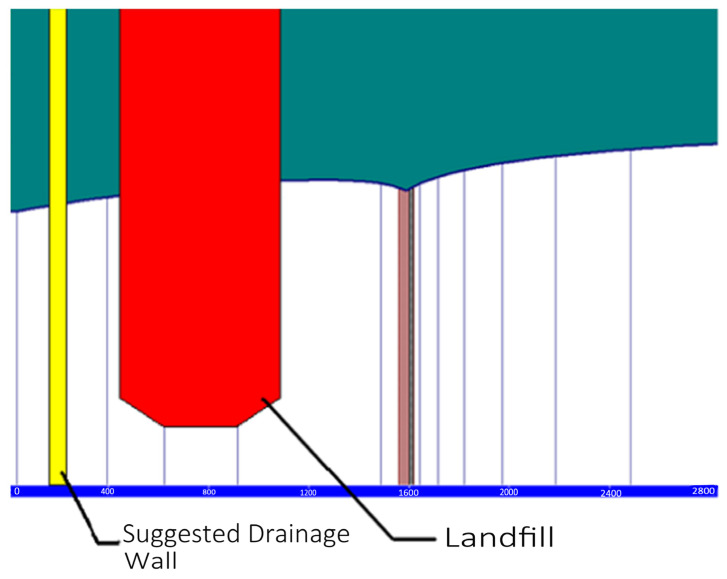
Recommended hydraulic barrier for preventing contaminants from being transported to Canadian territory from the CFSWMA landfill.

BOD is a good indicator of the impact of leachate from the CFSWMA landfill. As shown in Figure 6, BOD from the CFSWMA landfill indicates that groundwater quality and water quality deteriorates rapidly, with BOD flowing directly downstream to the north of the CFSWMA landfill. If a hydraulic (drainage) barrier is placed along the downstream gradient of the landfill, the potential effects of contaminants in the leachate can be always prevented.

BOD is an indirect measure of organic matter in water, reflecting the degree of organic pollution in landfill leachate. The transport of organic pollutants is influenced by several soil properties, including surface area, pH, cation exchange capacity, organic carbon content, and porosity. The natural attenuation of organic matter occurs primarily through dispersion and biodegradation [64]. In this study, soil organic carbon played a significant role in BOD adsorption, while BOD itself was found to be readily biodegradable. However, in the third subsurface layer, the limited oxygen and microbial activity substantially reduce biodegradation efficiency, extending the persistence of BOD in deeper strata.

Modeling results indicate that leachate from the CFSWMA landfill has not caused a significant impact on Canadian territory over a 20-year period following the potential leakage. Nevertheless, if the leakage is not repaired promptly, continuous effluent discharge could lead to pollutant accumulation in groundwater, thereby altering the overall risk profile.

In contrast, heavy metals such as Cu^2+^, Pb^2+^, and Zn^2+^ are non-biodegradable and migrate primarily through adsorption and retardation processes. Although the sandy till underlying the site shows low metal sorption capacity, long-term accumulation may still result in serious ecological and public health risks [65]. By comparison, conservative anions migrate almost entirely with groundwater and exhibit minimal toxicity.

Based on the AHP-based risk weighting and plume behavior comparison, BOD was selected as the key simulation indicator because it exhibits intermediate mobility, degradability, and risk characteristics, effectively representing the overall migration and attenuation behavior of landfill leachate contaminants while providing a realistic basis for predicting long-term environmental risks.

Moreover, in the future, energy usage and allocation can be further optimized, such as through measures to increase recycling, which can reduce W*_total_* to 0.25. In the future, 30% collaborative emission reduction can be achieved.

## 5. Conclusions

Taking the CFSWMA landfill as a case study, this research applied the coupled MODFLOW–EEMMS–MCM modeling framework to simulate groundwater flow, pollutant transport, and probabilistic environmental risk. BOD was selected as the representative indicator due to its moderate sorption, biodegradability, and ability to capture both migration and natural attenuation processes in landfill leachate.

The three-dimensional simulations revealed that non-sorptive chemicals are primarily governed by groundwater flow and are unlikely to affect surface water after 20 years due to biodegradation, while sorptive pollutants exhibit delayed migration influenced by retardation and adsorption effects. Some recommendations were made to reduce the potential transport of pollutants for better site remediation. Using this integrated risk assessment method, the adverse effects on the environment caused by all other kinds of chemical substances could also be easily modeled temporally and spatially. The most significant effects of leach solutions were found to be detectable and comparable to future regulatory standards.

Furthermore, mass transport simulation results show that the distribution of non-adsorbed chemicals is mainly affected by groundwater flow and will not pollute surface water after 20 years due to biodegradation. As for adsorptive pollutants, their transport in groundwater will be affected by many factors such as the retardation factor, sorption, and groundwater flow, etc.

Finally, the integration of energy balance modeling with environmental risk assessment established a dual evaluation framework that quantifies both pollutant behavior and energy consumption. This approach enhances predictive reliability and provides practical guidance for optimizing remediation design and developing sustainable solid waste management and reclamation strategies that balance ecological safety and energy efficiency.

## Figures and Tables

**Table 1:** Comparison of multimedia risk assessment models.

Model Name	Simple Introduction	Developer
A Multimedia Total Exposure Model for Hazardous Waste Sites (CalTOX)	Mainly models the risk value of soil pollution to the recipient	Lawrence Berkeley National Laboratory
Multimedia Environmental Pollutant Assessment System (MEPAS)	Mainly estimates environmental chronic diseases due to exposure	Pacific Northwest National Laboratory
The Multimedia Contaminant Fate, Transport, and Exposure Model (MMSOILS)	Mainly estimates the release of chemical contaminants from hazardous waste sites	USEPA Office of Research and Development
Multimedia, Multipathway, Multireceptor Risk Assessment (3MRA)	Mainly estimates the different exposures caused by pollutants	USEPA Office of Research and Development Office of Solid Waste
Extended Environmental Multimedia Modeling Analysis System (EEMMS)	The developed EEMMS can model air, landfill, unsaturated zones, and groundwater zones in 2D or 3D using the finite element method.	[36,44]

**Table 2:** Hydrological characteristics and contaminant sources of the CFSWMA landfill.

Layer	Bulk Density (kg/m^3^)	Porosity (n) %	Specific Storage (Ss) 1/ft	Specific Yield (Sy) 1/ft	Hydraulic Conductivity (K) ft/day	Dispersion (ft)
Upper glacial till	2200	20	3 × 10^−5^	0.055	1.86 × 10^−3^	40
Lower glacial till	2200	20	3 × 10^−5^	0.055	1.24 × 10^−4^	40
Total overburden	1700	20	3 × 10^−5^	0.082	1.53 × 10^−3^	46

**Table 3:** Water table in four directions at the CFSWMA landfill.

Surface Head (ft)	Depth (ft)	Top Elevation (ft)	Bottom Elevation (ft)
250.23	19	242.73	232.73
245.37	26.4	229.17	219.17
247.69	58	202.69	192.69
248.78	51	207.78	197.78

**Table 4:** Constructed judgment matrix.

Z	Heavy Metal	Organic	Inorganic
Heavy metal	1	5	7
Organic	1/5	1	3
Inorganic	1/7	1/3	1

**Table 5:** Column normalization and consistency check.

	Heavy Metal	Organic	Inorganic	W	AW
Heavy metal	0.7447	0.7895	0.6364	0.7235	2.2726
Organic	0.1489	0.1579	0.2727	0.1932	0.5879
Inorganic	0.1064	0.0526	0.0909	0.0833	0.2511

**Table 6:** Contaminant source characteristics.

Contaminant	Concentration (mg/L)	Kd (L/mg)
BOD	1000	N/A

**Table 7:** Comparative analysis of energy consumption reduction per unit risk in different environmental media.

Type	Energy Consumption (MJ/m^3^)	Standard Deviation	Sample Capacity	Resources
Soil	2.41 ± 0.37	0.29	127	[60]
Air	0.83 ± 0.12	0.09	89	[61]
Water	1.52 ± 0.21	0.17	104	[62]

## Data Availability

The data used and/or analyzed during the current study are available from the corresponding author on reasonable request.
